# Colistin-resistant Pseudomonas aeruginosa clinical strains with defective biofilm formation

**DOI:** 10.3205/dgkh000328

**Published:** 2019-10-10

**Authors:** Leila Azimi, Abdolaziz Rastegar Lari

**Affiliations:** 1 Pediatric Infections Research Center, Research Institute of Children’s Health, Shahid Beheshti University of Medical Sciences, Tehran, Iran; 2 Department of Microbiology, Iran University of Medical Sciences, Tehran, Iran

**Keywords:** Pseudomonas aeruginosa, colistin resistance, biofilm

## Abstract

**Aim:** Colistin is the only effective antibiotic in some cases of *Pseudomonas aeruginosa* resistance to all tested antibiotics, even carbapenem. On the other hand, biofilm formation is one of the antibiotic resistance mechanisms in this bacterium. The aim of this study was to examine biofilm formation in colistin-resistant *P. aeruginosa* for the first time.

**Method:** Two groups of *P. aeruginosa* were included in this study: 1) colistin-resistant and 2) colistin-susceptible.

Biofilm formation was determined in these groups using the micro-tube test well as PCR to detect the genes involved in biofilm formation (*ppk* and *molA*). The plasmids for colistin resistance, *mcr-1* and *mcr-2*, were also determined. *P. aeruginosa* ATCC 27853 was used as a control for all tests.

**Results:** Strong biofilm formation was observed only in colistin-susceptible strains, and *ppk* and *modA* were not detected in colistin-resistant strains. The control strain *P. aeruginosa* ATCC 27853 possesses *ppk* and *modA* and is categorized as a strong biofilm formation group. According to the results of this study, colistin resistance is associated with defective biofilm formation, as reported by other studies on *Acinetobacter baumannii*.

## Zusammenfassung

**Zielsetzung:** In einigen Fällen ist Colistin das einzige noch wirksame Antibiotikum, wenn *Pseudomonas aeruginosa* resistent gegen alle getesteten Antibiotika einschließlich Carbapenem ist. Andererseits ist die Biofilmbildung ein wichtiger Resistenzmechanismus dieses Bakteriums. Zielsetzung der Studie war daher die Untersuchung der Biofilmbildung Colistin-resistenter Stämme von *Pseudomonas aeruginosa*.

**Methode:** In die Studie wurden Colistin-empfindliche und -resistente Stämme eingeschlossen. Die Biofilmbildung wurde in Mikrotiterplatten bestimmt. Zugleich wurden die in die Biofilmbildung involvierten Gene *ppk* and *molA* mittels PCR bestimmt. Die Plasmide für die Colistinresistenz *mcr-1* und *mcr-2* wurden ebenfalls bestimmt.

**Ergebnisse:** Starke Biofilmbildung wurde nur bei Colistin-sensiblen Stämmen beobachtet. ppk und modA konnten nicht in Colistin-resistenten Stämmen detektiert werden. Der als Kontrollstamm untersuchte *P. aeruginosa* ATCC 27853 enthielt ebenfalls *ppk* and *modA* und ist daher zur starken Biofilmbildung fähig. Ein ähnlicher Zusammenhang zwischen Colistinresistenz und fehlender Biofilmbildung wurde in anderen Studien bei *Acinetobacter baumannii* nachgewiesen.

## Introduction

Multi-drug resistant (MDR) *Pseudomonas aeruginosa* strains are increasingly isolated from clinical specimens worldwide, which is cause for global concern [1], [2], [3], [4]. In some cases, only colistin remains as an effective antibiotic. However, this is problematic due to the nephrotoxicity of colistin and resistance to it, which can increase morbidity and mortality especially in immunosuppressed patients (e.g, hospitalized persons) [1], [2], [3], [4], [5], [6]. Colistin-resistant *P. aeruginosa* can be related to two chromosomal mutations: 1) modification of lipid A and 2) loss of LPS [7]. 

Dafopoulou et al. showed a loss of 47,969 bp genomic regions containing some genes like *ppk* and *modA*, which have been previously related to biofilm production in Enterobacteriaceae and *Pseudomonas* by whole genome mapping [7]. Thus, resistance to colistin can be associated with defective biofilm formation in *Pseudomonas*. Biofilm formation is one of the antibiotic-resistance mechanisms in *P. aeruginosa*, and can lead to cross-resistance based on the low penetration of antibiotics into the bacterial community after biofilm formation, and the appearance of MDR strains [2], [8].

Alarmingly, in 2016, first reports of plasmid-borne colistin resistance associated with *mcr-1* and *mcr-2* genes were published [9], [10]. The appearance of plasmid-borne colistin resistance is very important because of horizontal transmission in the bacterial population [9], [10]. The aim of this study was twofold:

1. to examine the relationship between colistin resistance and deficient biofilm formation in clinical *P. aeruginosa* strains, and
2. to test for *mcr-1* and *mcr-2* genes in colistin-resistant strains.

## Materials and methods

### Bacterial isolates

Two groups of colistin-resistant and colistin-susceptible strains of *P. aeruginosa* included 25 strains in each group. These were collected from blood culture and burn wound infections in pediatric and adult patients from two teaching hospitals, in Tehran. Collected strains were identified by conventional biochemical and microbiological tests, e.g. oxidase, TSI, lysine decarboxylase. Resistance to colistin was determined by the disc-diffusion agar method according to CLSI 2015 [11]. 

### Antibiotic susceptibility testing

Antibiotic susceptibility was tested using the Kirby Bauer disc-diffusion method according to CLSI guideline 2015 [11] against ceftazidime (30 µg), cefepime (30 µg), imipenem (10 µg), ticarcillin (75 µg), piperacillin (100 µg), piperacillin-tazobactam (100/10 µg), ciprofloxacin (5 µg), gentamicin (10 µg), tobramycin (10 µg) and amikacin (10 µg). Antibiotic discs used in this study were purchased from MAST (Mast Diagnostics, UK). *P. aeruginosa* ATCC 27853 was used as the control strain in the antibiotic susceptibility testing. Resistance and susceptibility to colistin were determined by minimum inhibitory concentration (MIC) using of the E. test strip in all strains.

### Phenotypic biofilm formation detection by micro-tube method

The micro-tube method, as described by Hassan et al., is a qualitative method for biofilm detection. A loop full of test organisms was inoculated in 1 mL of trypticase soy broth with 1% glucose in test tubes. The micro-tubes were incubated at 37°C for 24 h. After incubation, the micro-tubes were decanted and washed with phosphate buffer saline (pH 7.3) and dried. The micro-tubes were then stained with crystal violet (0.1%), and excess stain was rinsed off with deionized water. Micro-tubes were dried in the inverted position. The micro-tube biofilm results were classified as one of three types relative to the results of the control strains: 1) strong/high biofilm formation, 2) moderate, and 3) weak according to the mass of visible film lining the wall of the micro-tubes [12]. 

### Molecular detection of ppk and modA genes

*ppk* and *modA* genes were amplified by PCR and specific primers (Table 1 (Tab. 1)) in both colistin-resistant and colistin-susceptible strains. The conditions of PCR have been described previously [2]. DNA was extracted using the boiling method. Positive detection was confirmed by Sanger sequencing. Isolates which showed a specific band after PCR and electrophoresis were sent for sequencing to Pishgam, Macrogen, Seoul, Korea. *P. aeruginosa* ATCC 27853 was used as reference strain. (Tab. 2)(Tab. 3)

### Detection of mcr-1 and mcr-2

*mcr-1* and *mcr-2* were detected in colistin-resistant strains by PCR and specific primers (Table 1 (Tab. 1)). The PCR product load was determined on 1% agarose and visualized by gel document.

## Results

In this cross-sectional study, 25 colistin-resistant and 25 colistin-susceptible isolates were collected. All colistin-resistant *P. aeruginosa* strains were isolated from blood culture, and the colistin-susceptible strains were collected from burn wound infections. Ciprofloxacin and gentamicin are the most effective antibiotics against colistin-resistant and colistin-susceptible strains, respectively (Table 2 (Tab. 2)).

### Micro-tube test

Biofilm formation was classified into three groups according to tube test results: A weak, B moderate, and C strong. 

**Figure 1:**
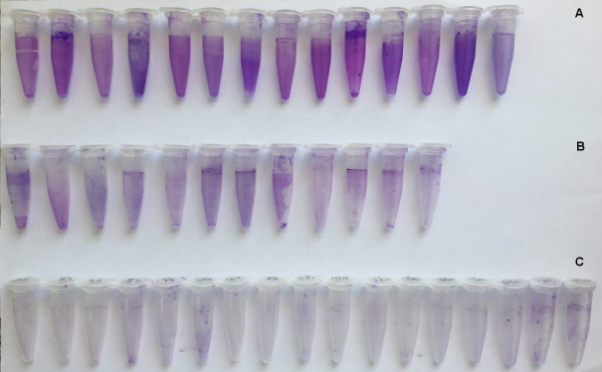
A: strong; B: moderate; C: weak

Strong biofilm formation was only observed in the colistin-susceptible strains (Table 3 (Tab. 3)). These three groups observed in the micro-tubes are depicted in Figure 1 (Fig. 1). *P. aeruginosa* ATCC 27853 possesses *ppk* and *modA*, and strong biofilm formation was observed.

### modA and ppk detection

**Figure 2:**
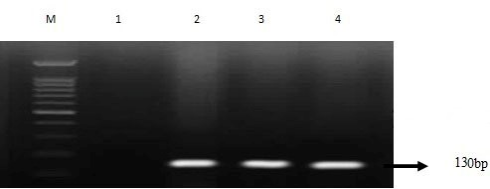
*modA*. M; marker 100 bp 1: negative control; 2: internal positive control; 3 and 4: positive strains

**Figure 3:**
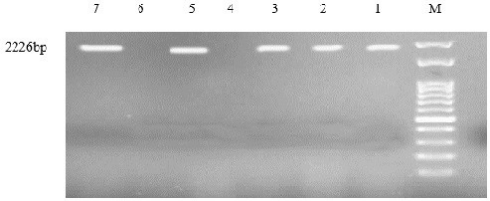
*ppk*. M; marker 100 bp 1: internal positive control; 2, 3, 5, 7: positive strains; 4: negative control; 6: negative strains

According to PCR and sequencing results, *modA* was detected in 23 (92%) of 25 and 17 (68%) of 25 colistin-susceptible strains (Figure 2 (Fig. 2), Figure 3 (Fig. 3)). But none of the colistin-resistant strains carried these two genes responsible for biofilm formation. The results of sequencing were confirmed by the positive results of the PCR assay.

### Mcr-1 and mcr-2 detection

These two plasmid-borne colistin-resistant genes were not detected in any of strains.

## Discussion

*P. aeruginosa* is one of a considerable number of Gram-negative bacteria that can cause nosocomial infections, especially in burn patients [1], [2], [3], [4], [5]. Monitoring *P. aeruginosa* in health care centers is very important, because it has great ability to survive in the hospital environment, its intrinsic antibiotic resistance mechanisms, and its potential for acquiring antibiotic-resistance genes [1], [2], [4]. Extensive drug-resistant (XDR) and MDR strains of *P. aeruginosa* can increase morbidity and mortality especially in hospitalized and immune-suppressed patients [1], [2], [3], [4], [5]. 

Carbapenems are the last line of defence in antibiotic therapy of ESBL-producing *P. aeruginosa* strains. Unfortunately, carbapenem-resistant strains have been reported worldwide, and in such cases, colistin remains the only effective antibiotic despite its nephrotoxicity [1], [2], [3], [4], [5], [6]. Up to 2016, all of the detected colistin-resistance mechanisms were attributed to chromosomal genes. Then, however, a plasmid-borne colistin-resistance gene was reported for the first time from China in 2016. *mcr-1* and *mcr-2* are both located on the plasmid and can perform horizontal transfer in bacteria [9], [10]. On the other hand, biofilm formation is one of the antibiotic-resistance mechanisms in *P. aeruginosa* [2], [8]. This Gram-negative bacterium can produce biofilm on the hospital surfaces and catheters, cause therapeutic complications and consequently prolongation of hospital stays, which finally impose increased health care costs for society and patients [8], [13], [14]. The results of a study in France in 2015 on *A. baumannii* indicated that chromosomal resistance to colistin is associated with the loss of 47,969 bp genomic regions containing *modA* and *ppk* (genes involved in biofilm formation in Pseudomonas), according to whole genome sequencing results [7]. Thus, colistin-resistant *A. baumannii* strains can exhibit defective biofilm formation [7]. The results of the current study confirmed this observation. The results of the micro-tube test indicated that biofilm formation was weak and moderate in 84% and 16% of colistin-resistant strains, respectively. In contrast, biofilm formation was strong and moderate in 52% and 32% of colistin-susceptible *P. aeruginosa*. *modA* and *ppk* detection confirmed this hypothesis, because none of the colistin-resistant strains carried both of these genes. On the other hand, all colistin-susceptible strains harbored at least one of these two biofilm formation genes, except two isolates.

## Conclusion

According to the results of this study, colistin resistance can be accompanied by defective biofilm formation, similar to the results of Defopoulou et al. on *A. baumannii*. Future studies on different antibiotic resistances and their combined effect will be very important and helpful in promoting more successful antibiotic therapy and thus better health, especially pediatric patients.

## Notes

### Funding 

The research reported in this publication was supported by Elite Researcher Grant Committee under award number [958699] from the National Institutes for Medical Research Development (NIMAD), Tehran, Iran.

### Competing interests

The authors declare that they have no competing interests.

## Figures and Tables

**Table 1:**
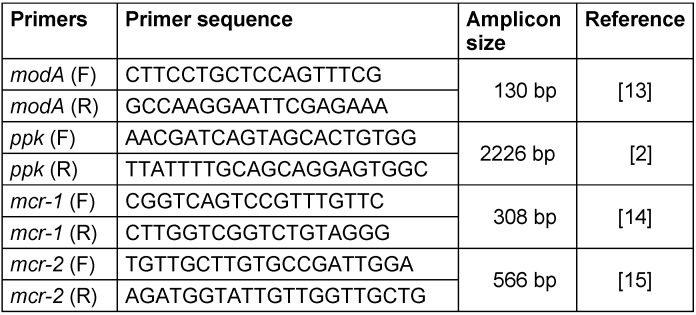
Primer sequences used in this study

**Table 2:**
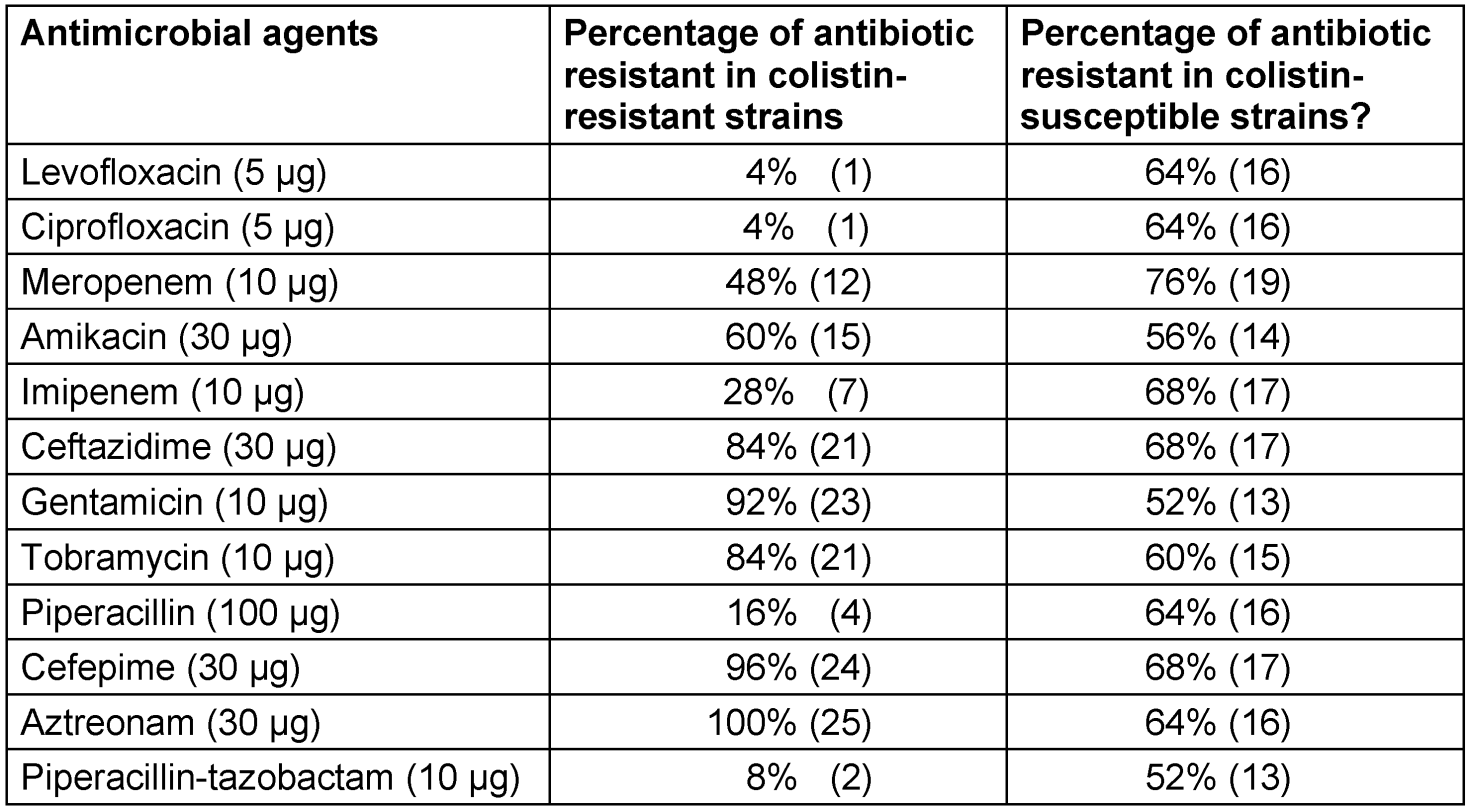
Percent resistance of antimicrobial agents in colistin-resistant and colistin-susceptible groups

**Table 3:**
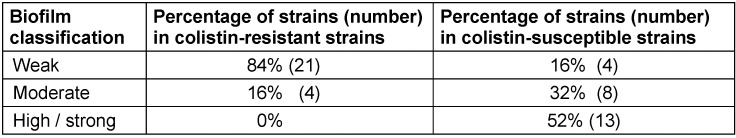
Biofilm formation observed in the micro-tube test in in colistin-resistant and colistin-susceptible groups
